# Maternal influenza vaccine strategies in Kenya: Which approach would have the greatest impact on disease burden in pregnant women and young infants?

**DOI:** 10.1371/journal.pone.0189623

**Published:** 2017-12-28

**Authors:** Meredith L. McMorrow, Gideon O. Emukule, David Obor, Bryan Nyawanda, Nancy A. Otieno, Caroline Makokha, Joshua A. Mott, Joseph S. Bresee, Carrie Reed

**Affiliations:** 1 Influenza Division, National Center for Immunization and Respiratory Diseases, Centers for Disease Control and Prevention (CDC), Atlanta, Georgia, United States of America; 2 United States Public Health Service, Rockville, Maryland, United States of America; 3 Centers for Disease Control and Prevention-Kenya Country Office, Nairobi, Kenya; 4 Kenya Medical Research Institute (KEMRI), Kisumu, Kenya; Pfizer Inc, UNITED STATES

## Abstract

**Background:**

Recent influenza surveillance data from Africa suggest an important burden of influenza-associated morbidity and mortality. In tropical countries where influenza virus transmission may not be confined to a single season alternative strategies for vaccine distribution via antenatal care (ANC) or semiannual campaigns should be considered.

**Methods:**

Using data on monthly influenza disease burden in women of child-bearing age and infants aged 0–5 months in Kenya from 2010–2014, we estimated the number of outcomes (illnesses, medical visits, hospitalizations, and deaths) that occurred and that may have been averted through influenza vaccination of pregnant women using: 1) a year-round immunization strategy through ANC, 2) annual vaccination campaigns, and 3) semiannual vaccination campaigns.

**Results:**

During 2010–2014, influenza resulted in an estimated 279,047 illnesses, 36,276 medical visits, 1612 hospitalizations and 243 deaths in pregnant women and 157,053 illnesses, 65,177 medical visits, 4197 hospitalizations, and 755 deaths in infants aged 0–5 months in Kenya. Depending on the mode of distribution and the vaccine coverage achieved, 12.8–31.4% of influenza-associated disease in pregnant women and 11.6–22.1% in infants aged 0–5 months might have been prevented through maternal influenza immunization. In this model, point estimates for influenza-associated disease averted through maternal vaccination delivered year-round in ANC or semiannually in campaigns were higher than vaccination delivered in a single annual campaign, but confidence intervals overlapped.

**Conclusions:**

Vaccinating pregnant women against influenza can reduce the burden of influenza-associated illness, hospitalization and death in both pregnant women and their young infants. Alternative immunization strategies may avert more influenza-associated disease in countries where influenza virus transmission occurs throughout the year.

## Introduction

Influenza remains a major cause of morbidity and mortality worldwide. While influenza immunization programs have been effective tools in reducing the disease burden in high-income countries [1, 2], few low middle- and low-income countries have robust national influenza vaccination programs [3]. Investments in influenza surveillance in many African countries during the last decade have confirmed a substantial influenza burden in the region, especially among persons in high-risk groups, such as young children, pregnant women, elderly and those with chronic medical conditions who are at increased risk of hospitalization and death from influenza virus infection [4–10]. Annual vaccination is the most effective method for preventing influenza virus infection and associated complications [11]. In 2012, the WHO Strategic Advisory Group of Experts (SAGE) on Immunization concluded that pregnant women are the highest priority among risk groups for seasonal influenza vaccination based upon “compelling evidence of substantial risk of severe disease in this group and evidence that seasonal influenza vaccine is safe and effective in preventing disease in pregnant women as well as their young infants, in whom disease burden is also high” [12]. Vaccination of pregnant women has been shown to provide protection to their infants against laboratory-confirmed influenza illness and hospitalization in the first months of life [13–19]. As a result, many countries have or are considering expanding influenza vaccination programs to include pregnant women or targeting pregnant women as part of new influenza immunization strategies [20, 21].

Although data are limited, the impact of influenza on adverse outcomes in pregnant women and children is likely to be as great, or greater in developing world settings as compared to more developed countries. A recent systematic review estimated that 99% of influenza-related deaths among children aged less than five years occur in resource-limited settings, and the incidence of severe acute lower respiratory tract infections among infants aged 0–11 months appears to be greater in low-resource settings [22]. In Kenya, higher prevalence of HIV, tuberculosis and other risk factors may further predispose pregnant women and their infants to severe outcomes from influenza infection [23–25]. Using data from population-based [26, 27] and health and demographic surveillance (HDS) [28] sites, we estimated the burden of influenza-associated illness among pregnant women and infants aged 0–5 months and the influenza outcomes that could be averted through influenza vaccination of pregnant women in Kenya.

## Materials and methods

A model was constructed using estimates of influenza disease burden, vaccine coverage, and vaccine effectiveness to estimate influenza outcomes that could have been averted through influenza vaccination of pregnant women in Kenya. Model inputs include data from studies that are human subjects research. These protocols require informed consent and are reviewed and approved by the Kenya Medical Research Institute and the U.S. Centers for Disease Control and Prevention. We compared the following strategies for vaccinating pregnant women: 1) a year-round immunization strategy in which vaccines are provided through antenatal clinics (ANC), 2) annual vaccination campaigns using Southern Hemisphere formulations of influenza vaccine (delivered in March and April), or 3) semi-annual vaccination (every 6 months) campaigns using both Northern and Southern Hemisphere vaccine formulations. Outcomes assessed included averted influenza cases, medical visits, hospitalizations, and deaths in pregnant women and their infants.

### Seasonality of influenza-associated illness

**Fig 1:**
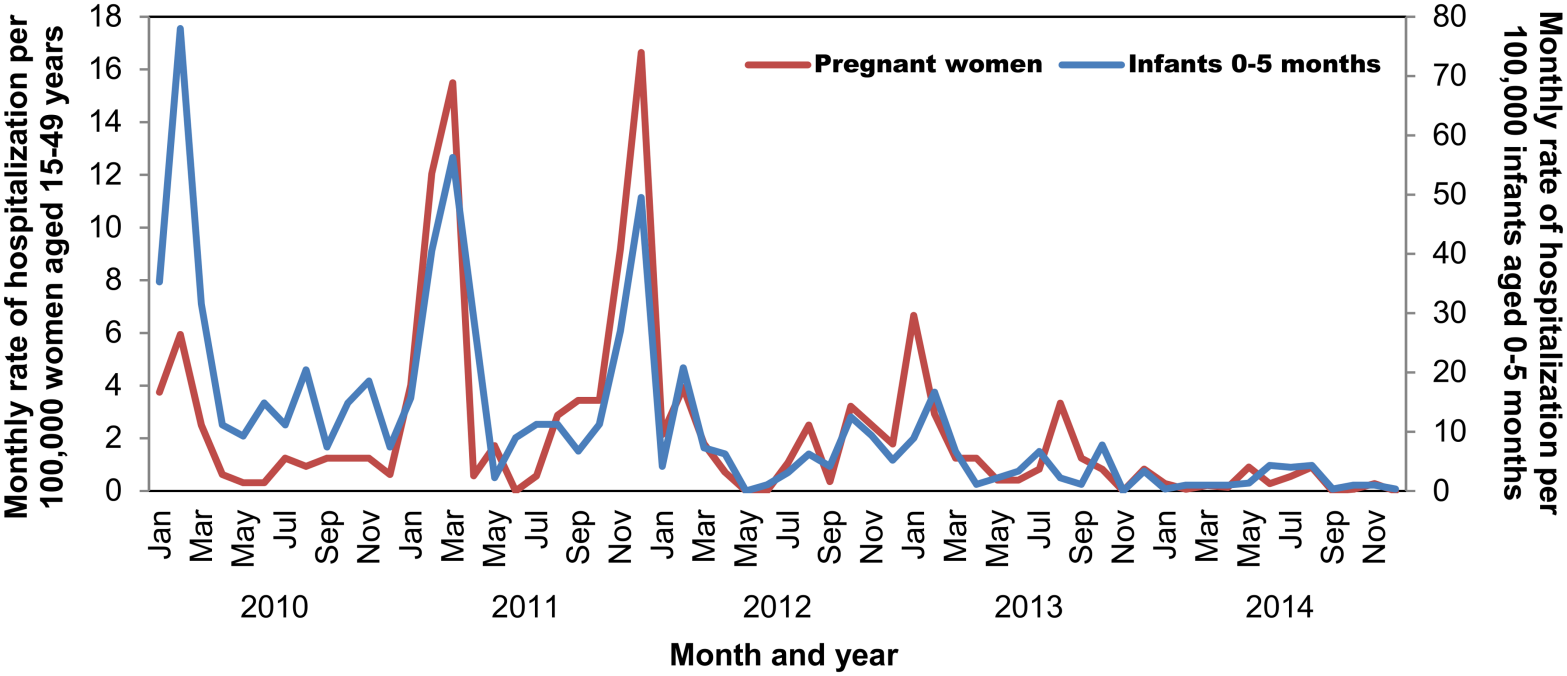
Monthly rate of influenza-associated hospitalization per 100,000 women aged 15–49 years or per 100,000 infants aged 0–5 months, Kenya, 2010–2014*.

During the period of analysis, influenza occurred year-round in Kenya, but seasonal peaks in disease were observed. Cases of influenza associated with the 2009 influenza A(H1N1) pandemic virus were first detected in Kenya in July 2009 [29], and spread rapidly causing high rates of hospitalization among infants aged 0–5 months and women aged 15–49 years in January–March 2010 (Fig 1). In 2011, there were 2 large peaks of influenza hospitalization in both women aged 15–49 years and infants from January-April and September-December. In 2012–2014, seasonality returned to the typical pattern of an early peak February-April followed by a second peak July-November [29].

### Rates of influenza-associated mortality and proportion of deaths outside the hospital

We used the 5-year average mortality rate in hospitalized women aged 15–49 years with a respiratory diagnosis from an HDS site in western Kenya [6.8% (95% CI 1.1–12.5%)], the respiratory admission rate among women aged 15–49 years, and the proportion of respiratory hospitalizations that tested positive for influenza viruses to estimate the influenza-associated mortality rate in hospitalized pregnant women (Table 1). We used the 5-year average mortality rate in hospitalized children aged 0–5 months with a respiratory diagnosis from the HDS site [2.4% (95% CI 0.2–4.6%)] and the proportion of respiratory hospitalizations that tested positive for influenza to estimate influenza-associated mortality in hospitalized young infants. Some persons with severe influenza are not hospitalized and die in the community. Verbal autopsy data on respiratory deaths in infants aged 0–5 months and women of childbearing age were used to estimate the proportion of influenza-associated deaths occurring outside the hospital. From HDS verbal autopsy data it was determined that the percentage of deaths in women aged 15–49 years occurring outside the hospital ranged from 46.2% to 66.7% per year during 2010–2012; data were not available for 2013–2014, so the 2012 estimate was applied to these years as it was the lowest and most conservative approximation. HDS verbal autopsy data estimate 73.5% to 91.2% of annual respiratory deaths in infants aged 0–5 months occurred outside the hospital during 2010–2012; likewise, data were not available for 2013–2014 so the 2012 estimate was used.

**Table 1:** Model inputs and data sources.

Model input	Data Source	Year(s)	Value(s)
**Kenya population estimates**	**Kenya Population and Housing Census 2009** http://www.knbs.or.ke/index.php?option=com_phocadownload&view=category&download=584:volume-1c-population-distribution-by-age-sex-and-administrative-units&id=109:population-and-housing-census-2009&Itemid=599	2009	
**• Women aged 15–49 years**			9,375,784
**• Infants 0–11 months**			1,221,937
**Population growth rate (average annual %)**	**UN Data** http://data.un.org/CountryProfile.aspx?crName=kenya	2010–2015	2.7
**General fertility rate per 1000 women aged 15–44 years**	**Kenya Demographic and Health Survey 2008–2009** http://www.dhsprogram.com/pubs/pdf/FR229/FR229.pdf	2006–2008	161
**Infant mortality rate per 1000 live births**	**Kenya Demographic and Health Survey 2008–2009**	2004–2008	52
**Mortality rate among respiratory admissions (%)**	**KEMRI Health and Demographic Surveillance**	2010–2014	
**• Women aged 15–49 years**			6.8 (95% CI 1.1–12.5)
**• Infants aged 0–5 months**			2.4 (95% CI 0.2–4.6)
**Proportion of respiratory deaths occurring outside of the hospital (%)**	**KEMRI Health and Demographic Surveillance**		
**• Women aged 15–49 years**		2010	66.7 (95% CI 29.9–92.5)
		2011	55.6 (95% CI 21.2–86.3)
		2012	46.2 (95% CI 19.2–74.9)
**• Infants aged 0–5 months**		2010	91.2 (95% CI 88.0–97.0)
		2011	83.7 (95% CI 72.7–88.5)
		2012	73.5 (95% CI 67.8–85.9)
**Annual influenza-associated hospitalization rate/100,000**	**KEMRI Population Based Infectious Disease Surveillance**	2010–2014	
**• Women aged 15–49 years**			39.9 (95%CI 25.3–62.9)
**• Infants aged 0–5 months**			129.4 (95% CI 45.8–365.7)
**Increased odds of influenza-associated hospitalization in pregnancy from 3 sites**	**KEMRI, Malawi-Liverpool-Wellcome Trust, and National Institute for Communicable Diseases, South Africa** [30]	2009–2015	2.9 (95% CI 1.0–12.25)
**Estimated number of cases per hospitalization**	**KEMRI Population Based Infectious Disease Surveillance**	2010–2014	
**• Women aged 15–49 years**			23
**• Infants aged 0–5 months**			16
**Proportion of illnesses that are medically attended (%)**	**KEMRI Population Based Infectious Disease Surveillance**	2010–2014	
**• Women aged 15–49 years**			13
**• Infants aged 0–5 months**			42
**Proportion of pregnant women with 2 or more antenatal care visits (%)**	**Kenya Demographic and Health Survey 2008–2009**	2008–2009	86
**Vaccine effectiveness (%)**	Estimated using 1-risk ratio (RR); RR estimated using random effects Mantel-Haenszel model of published randomized controlled trials of inactivated influenza vaccine in pregnant women (see text for references)		
**• Women aged 15–49 years**		2011–2014	61.0% (95% CI, 43.2–73.2%) n = 6430
**• Infants aged 0–5 months**		2008–2014	56.4% (95% CI 38.6–69.0) n = 6658
**Vaccine coverage with 2 doses of tetanus toxoid among pregnant women (%)**	**Kenya Demographic and Health Survey 2008–2009**	2008	55

### Rates of influenza-associated hospitalization

We used data on the rate of influenza-associated acute lower respiratory tract infection hospitalizations in women aged 15–49 years and infants aged 0–5 months from a HDS site in western Kenya and applied this rate to the number of pregnant women and infants aged 0–5 months in the HDS site catchment area. Because limited data were available on disease burden among pregnant women in Kenya, we used hospitalization rates among women of childbearing age as a proxy. There are limited data on the risk of influenza-associated hospitalization among pregnant compared to non-pregnant women in Africa. We did not make an adjustment for the sensitivity of influenza testing by rRT-PCR or influenza-associated non-respiratory illnesses in pregnant women or infants. The average annual hospitalization rate over the 5-year study period was 39.9 (95% CI 25.3–62.9) per 100,000 women aged 15–49 years and 129.4 (95% CI 45.8–365.7) per 100,000 infants aged 0–5 months.

### Rates of medically attended (MA) illness

We estimated the number of medically attended influenza illnesses in Kenyan pregnant women and their infants by applying a ratio of medically attended cases to hospitalizations determined by dividing the total number of medically attended (MA) ILI and SARI attributable to influenza in women aged 15–49 years or infants aged 0–5 months from population-based surveillance by the hospitalization rate in women aged 15–49 years or infants aged 0–5 months. In this study our medically attended case-hospitalization ratio was 22.5 for women aged 15–49 years and 15.5 for infants aged 0–5 months.

### Rates of influenza illness

We estimated the number of influenza illnesses in Kenyan pregnant women and their infants by dividing the medically attended illness rates by the proportion of ILI and SARI that were medically attended. From population-based surveillance, the proportion of all ILI and SARI that were medically attended was 13.0% for women aged 15–49 years and 41.5% for infants aged 0–5 months. We assumed that protection against repeat infection conferred through influenza illness was complete and would last throughout the pregnancy (against maternal infection) or the first 6 months of life (against infant infection).

### Influenza vaccine coverage

The most recent estimate of vaccination coverage of pregnant women in Kenya with two doses of tetanus toxoid is 55.0% [31], which is recommended for all pregnant women in Kenya and administered year-round in ANC. Assuming that influenza vaccination in ANC could achieve similar coverage rates, approximately 4.6% of pregnant women would be vaccinated monthly in the year-round model. For the first comparison, we assumed 55% of all women who become pregnant in a year would be vaccinated in either annual or semiannual campaigns. We assumed that protection conferred through influenza vaccination would last through the remainder of the pregnancy and the infant’s first 6 months of life.

After comparing the three vaccination strategies assuming all achieved 55% coverage of all women who become pregnant in a year, we made additional comparisons of hospitalizations averted by the different strategies with the following assumptions: 1. If 86% of women have 2 or more antenatal care visits [31] and 80% of them received an influenza vaccine, then vaccine coverage through ANC could reach 69%; 2. If women are unlikely to identify as pregnant until they have completed the first trimester of pregnancy then approximately 50% of all women who will be pregnant during a year would identify as such in any given month and 86% of them would have 2 or more ANC visits. If 80% of the women that present in a month could be reached with an annual campaign then an annual campaign would likely achieve 34% vaccine coverage; 3. Making the same assumptions as for a single campaign, semiannual campaigns could potentially achieve 69% vaccine coverage.

### Influenza vaccine effectiveness

Vaccine effectiveness (VE) was estimated using 1-risk ratio (RR) (Table 1) where the RR was estimated using a random effects Mantel-Haenszel model of published randomized controlled trials (RCTs) of inactivated influenza vaccine in pregnant women (2 RCTs) and their infants (3 RCTs) using the metan command in Stata version 14 (StataCorp, College Station, TX) [13, 18, 19].

In September 2009, influenza A(H1N1)pdm09 virus began circulating in Kenya. Monovalent pandemic vaccine was not available in Kenya until April 2010. During this 8 month period, 85% of influenza viruses identified in routine and population-based surveillance in Kenya were influenza A(H1N1)pdm09. To reflect the reduced vaccine effectiveness of the available seasonal vaccine against pandemic influenza A(H1N1) we assumed that VE was 0% from January to April 2010. This was done to ensure that we did not overestimate the potential impact of the available seasonal vaccination during the initial stages of the pandemic. In 2010 the annual campaign was delayed by 2 months to May-June to more accurately reflect pandemic vaccine availability.

### Estimating averted burden

We estimated the averted burden of influenza-related outcomes that could be achieved with prenatal influenza vaccination in several steps, similar to the methodology used in the United States[1]. First we estimated the number of influenza-associated outcomes that occurred in each month using data from population-based surveillance for influenza [26, 27, 32] and health and demographic surveillance system (HDSS) [28] in Kenya, as described above. We used these rates as the baseline for disease occurrence in the absence of influenza vaccination, as influenza vaccine is not regularly used among pregnant women in Kenya. To these baseline rates, we then applied data on potential influenza vaccine coverage and influenza vaccine effectiveness for pregnant women and their infants aged 0–5 months to calculate a second estimate of disease burden if vaccination had been available. The burden averted by vaccination was estimated by the difference between outcomes with vs. without vaccination. Each year’s model was stratified by month to accommodate time-sensitive patterns of vaccination coverage and disease occurrence. This process was repeated for pregnant women and infants aged 0–5 months using the three vaccination strategies defined above. Confidence intervals for the reported results were estimated using a Monte Carlo algorithm, drawing values from sampling distribution of the input variables used in the model including disease rates and VE against influenza in the pregnant woman and infant.

### Estimating the prevented fraction

The number of outcomes averted each year not only depends directly on vaccination coverage and effectiveness, but also depends on the influenza attack rate–i.e., years with high attack rates will produce a higher number of averted outcomes assuming the same coverage rate and VE. Therefore, we present averted influenza burden for each vaccination strategy in absolute numbers and as the prevented fraction. The prevented fraction is defined in this analysis as the proportion of outcomes averted through vaccination out of potential outcomes in the absence of vaccination. Each year, the prevented fraction is the same for all outcomes (cases, medically-attended cases, and hospitalizations) because the number of cases and MA cases were calculated as proportions of the estimated number of hospitalizations each year using data on reported ILI and SARI, and medically-attended ILI and SARI from population-based surveillance.

### Number needed to vaccinate

We further calculated the number of pregnant women that would need to be vaccinated in order to prevent one maternal or infant hospitalization, and one maternal or infant death by dividing the total number of women vaccinated per year by the number of averted episodes (hospitalizations, deaths).

### Sensitivity analysis

In order to assess the impact of higher rates of influenza-associated hospitalization in pregnant women, we used published and data from 3 countries (Kenya, Malawi, and South Africa)[30] to estimate the increased odds of influenza-associated hospitalization among pregnant women. We multiplied the current hospitalization rates by this ratio and assessed the potential impact on averted disease burden.

## Results

### Baseline estimates of influenza illness, medical visits, hospitalizations and deaths

During 2010–2014, we estimate that influenza virus infections among pregnant women caused 279,047 illnesses, 36,276 medical visits, 1612 hospitalizations, and 243 maternal deaths (Table 2). We estimate that during 2010–2014, among Kenyan infants aged 0–5 months 157,053 illnesses, 65,177 medical visits, 4197 hospitalizations, and 755 deaths may have been caused by influenza virus infections. Of the infant deaths, 422 (56%) were estimated to have occurred in 2010 during the pandemic period.

**Table 2:** Baseline estimates of influenza illnesses, medical visits, hospitalizations and deaths among pregnant women and infants aged 0–5 months in Kenya, 2010–2014.

	Pregnant Women				Infants aged 0–5 months			
Year	Illnesses (95% CI)	Medical Visits (95% CI)	Hospitalizations (95% CI)	Deaths (95% CI)	Illnesses (95% CI)	Medical Visits (95% CI)	Hospitalizations (95% CI)	Deaths (95% CI)
**2010**	40,235 (19,852–60,562)	5,231 (2581–7873)	232 (115–350)	47 (6–101)	57,897 (42,334–73,354)	24,027 (17,569–30,442)	1,547 (1131–1960)	422 (44–849)
**2011**	144,584 (82,597–206,312)	18,796 (10,738–26,821)	835 (477–1192)	127 (18–263)	61,263 (45,384–77,089)	25,424 (18,834–31,992)	1,637 (1213–2060)	241 (24–481)
**2012**	42,413 (20,934–63,709)	5,514 (2721–8282)	245 (121–368)	31 (4–66)	18,638 (11,709–25,545)	7,735 (4859–10,601)	498 (313–683)	45 (4–93)
**2013**	43,545 (21,847–65,313)	5,661 (2840–8491)	252 (126–377)	32 (4–67)	14,318 (7118–21,502)	5,942 (2954–8923)	383 (190–575)	35 (3–75)
**2014**	8,271 (2280–14,304)	1,075 (296–1860)	48 (13–83)	6 (0–14)	4,936 (2461–7408)	2,048 (1021–3075)	132 (66–198)	12 (1–26)
**Total**	**279,047 (147,510–410,200)**	**36,276 (19,176–53,326)**	**1,612 (852–2370)**	**243 (33–512)**	**157,053 (109,006–204,898)**	**65,177 (45,238–85,032)**	**4,197 (2913–5476)**	**755 (76–1525)**

### Comparison of influenza-associated disease potentially averted by different vaccination strategies using equal vaccine coverage (55%)

#### Maternal outcomes averted

**Fig 2:**
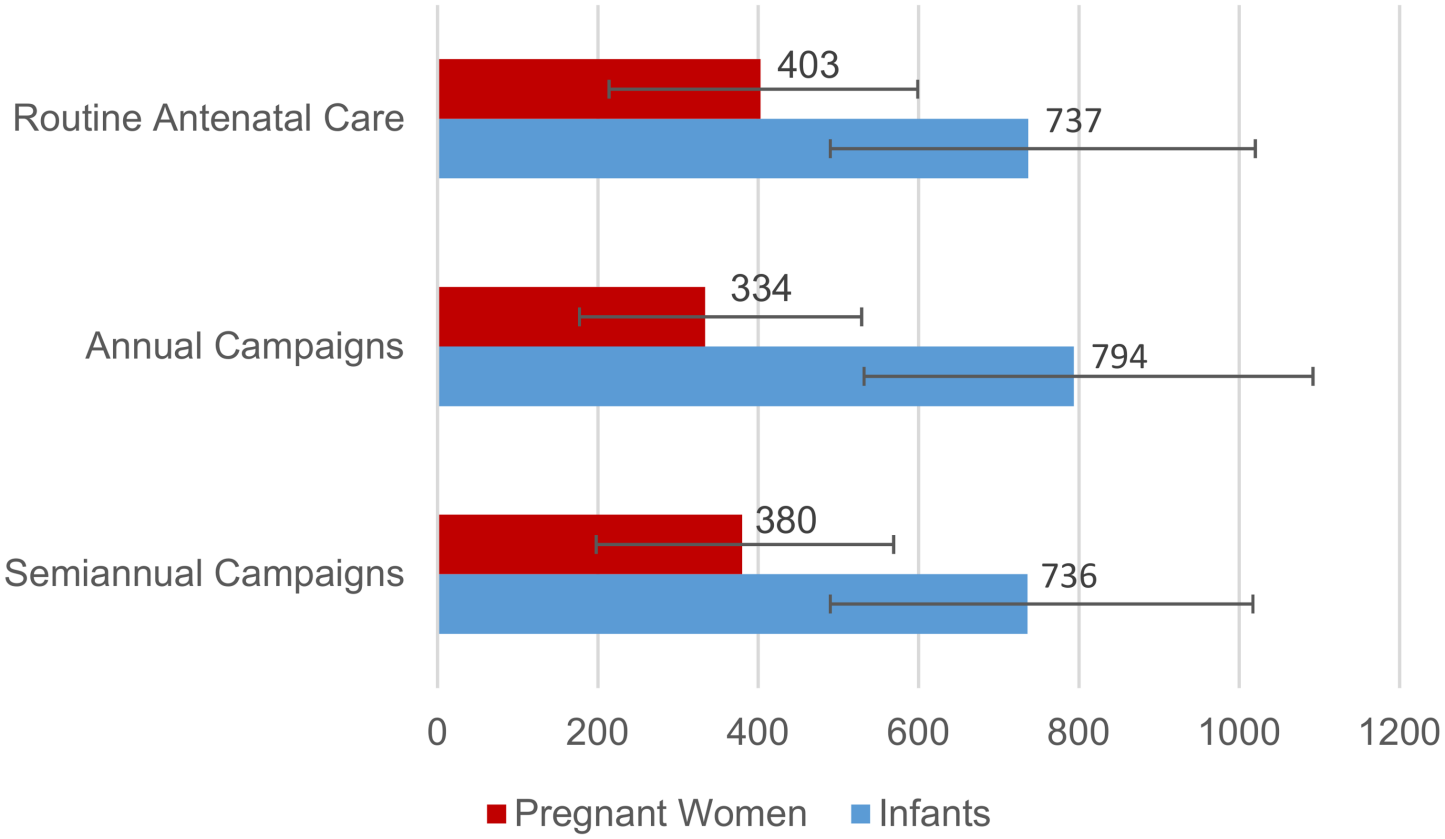
Estimated influenza-associated hospitalizations potentially averted through influenza vaccination with 55% coverage by strategy, Kenya, 2010–2014.

Delivery of influenza vaccine through routine antenatal care with 55% coverage might have averted 69,780 influenza-associated illnesses, 9071 medical visits, 403 hospitalizations and 58 deaths in pregnant women over the 5-year study period (Table 3, Fig 2). The averted fraction was 25.0% (range by year 5.8–29.2%). Delivery of influenza vaccine through annual vaccination campaigns in March-April (May-June in 2010) with 55% coverage might have averted 57,799 influenza-associated illnesses, 7514 medical visits, 334 hospitalizations and 58 deaths in pregnant women over the 5-year study period. The averted fraction was 20.7% (range by year 9.8%-35.9%). Delivery of influenza vaccine through semiannual vaccination campaigns in March-April (May-June in 2010) and Sept-Oct with 55% coverage might have averted 65,828 illnesses, 8558 medical visits, 380 influenza-associated hospitalizations and 55 deaths in pregnant women over the 5-year study period. The averted fraction was 23.6% (range by year 7.2–30.3%).

**Table 3:** Comparison of influenza-associated disease burden potentially averted through different maternal immunization strategies, Kenya, 2010–2014.

	Pregnant Women					Infants aged 0–5 months				
Immunization Strategy	Illnesses Averted (95% CI)	Medical Visits Averted (95% CI)	Hospitalizations Averted (95% CI)	Deaths Averted (95% CI)	Averted fraction,% (range by year)	Illnesses Averted (95% CI)	Medical Visits Averted (95% CI)	Hospitalizations Averted (95% CI)	Deaths Averted (95% CI)	Averted fraction,% (range by year)
**Assuming 55% coverage for all strategies**										
**Routine Antenatal Care**	69,780 (37,097–103,671	9071 (4823–13,477)	403 (214–599)	58 (8–123)	25.0 (5.8–29.2)	27,575 (18,352–38,158)	11,444 (7616–15,835)	737 (490–1020)	98 (10–204)	17.6 (3.3–30.1)
**Annual Campaign**	57,799 (30,579–91,575)	7514 (3975–11,905)	334 (177–529)	58 (7–108)	20.7 (9.8–35.9)	29,712 (19,894–40,862)	12,331 (8256–16,958)	794 (532–1092)	113 (11–234)	18.9 (6.3–32.3)
**Semiannual Campaign**	65,828 (34,250–98,496)	8558 (4453–12,804)	380 (198–569)	55 (7–117)	23.6 (7.2–30.3)	27,529 (18,343–38,057)	11,424 (7612–15,794)	736 (490–1017)	100 (10–208)	17.5 (4.2–30.0)
**Assuming different coverage per strategy**										
**Routine Antenatal Care (69% coverage)**	87,690 (46,554–130,097)	11,400 (6052–16,913)	507 (269–752)	73 (10–155)	31.4 (7.2–36.7)	34,692 (23,009–47,844)	14,397 (9549–19,855)	927 (615–1279)	123 (12–256)	22.1 (4.1–37.8)
**Annual Campaign (34% coverage)**	35,650 (18,887–56,560)	4635 (2455–7353)	206 (109–327)	30 (4–67)	12.8 (6.1–22.2)	18,257 (12,321–25,307)	7576 (5113–10,502)	488 (329–676)	69 (7–145)	11.6 (3.9–19.9)
**Semiannual Campaign (69% coverage)**	82,719 (42,984–123,613)	10,753 (5588–16,070)	478 (248–714)	69 (9–146)	29.6 (9.0–38.1)	34,629 (22,999–47,719)	14,371 (9544–19,803)	925 (615–1275)	126 (12–261)	22.0 (5.3–37.7)

#### Infant outcomes averted

Delivery of influenza vaccine through routine antenatal care with 55% coverage might have averted 27,575 influenza-associated illnesses, 11,444 medical visits, 737 hospitalizations, and 98 deaths in infants aged 0–5 months over the 5-year study period (Table 3, Fig 2). The averted fraction in infants aged 0–5 months was 17.6% (range by year 3.3–30.1%). Delivery of influenza vaccine through annual vaccination campaigns in March-April (May-June in 2010) with 55% coverage might have averted 29,712 influenza-associated illnesses, 12,331 medical visits, 794 hospitalizations, and 113 deaths in infants aged 0–5 months over the 5-year study period. The averted fraction in infants aged 0–5 months was 18.9% (range by year 6.3–32.3%). Delivery of influenza vaccine through semiannual vaccination campaigns in March-April (May-June in 2010) and Sept-Oct with 55% coverage might have averted 27,529 illnesses, 11,424 medical visits, 736 influenza-associated hospitalizations and 100 deaths in infants aged 0–5 months over the 5-year study period. The averted fraction was 17.5% (range by year 4.2%-30.0%).

#### Number needed to vaccinate

The number needed to vaccinate to prevent one maternal or infant hospitalization was similar for all three strategies (ANC– 3944, annual campaign– 3986 and semiannual campaigns– 4028). Likewise, the number needed to vaccinate to prevent one maternal or infant death was similar for all three strategies (ANC– 28,819, annual campaigns– 27,924 and semiannual campaigns– 29,004).

### Comparison of influenza-associated disease potentially averted by different vaccination strategies using different assumptions on vaccine coverage

#### Maternal outcomes averted

**Fig 3:**
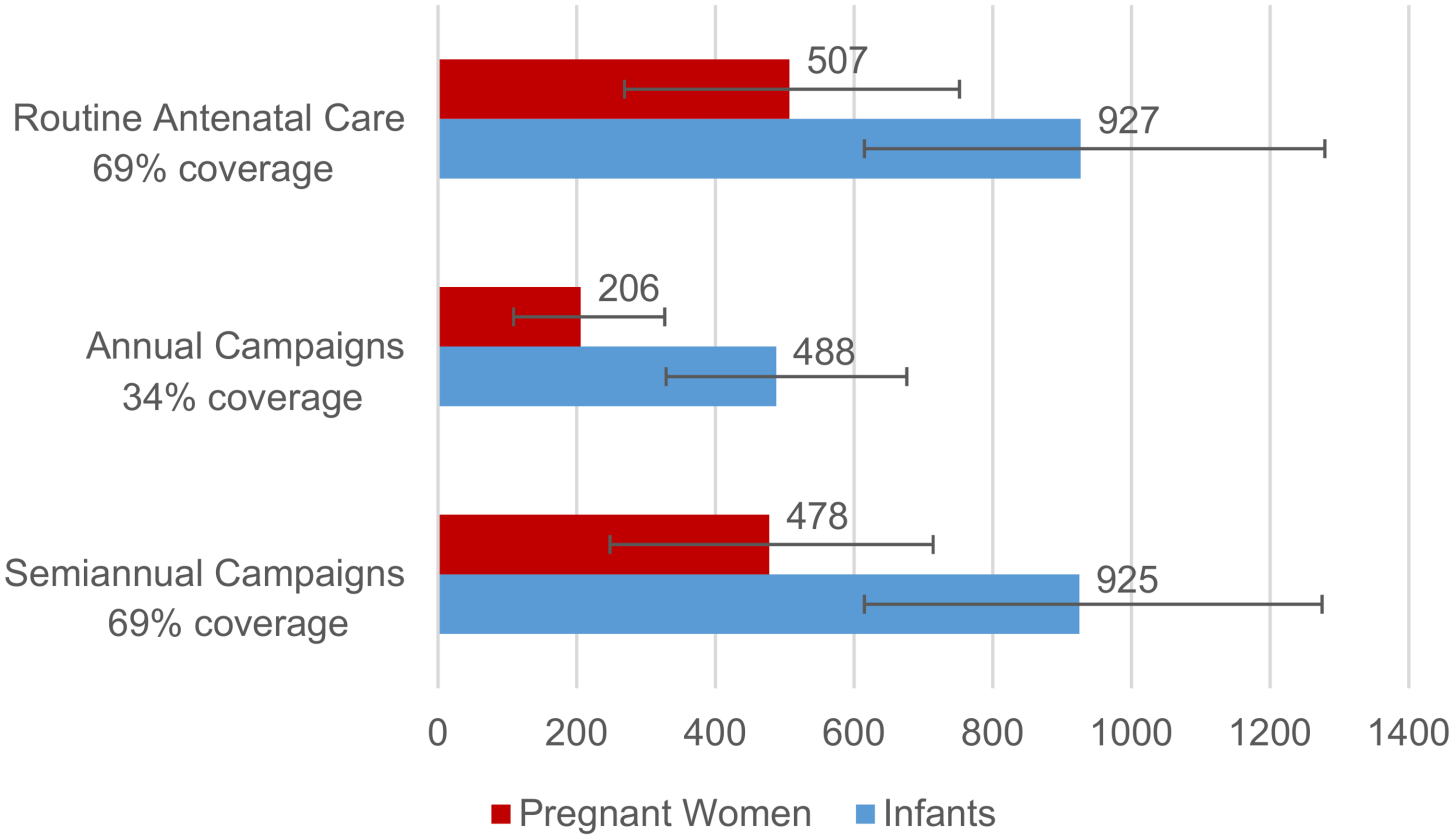
Estimated influenza-associated hospitalizations potentially averted through influenza vaccination with different coverage assumptions by strategy, Kenya, 2010–2014.

Delivery of influenza vaccine through routine antenatal care with 69% coverage might have averted 87,690 illnesses, 11,400 medical visits, 507 influenza-associated hospitalizations and 73 deaths in pregnant women over the 5-year study period (Table 3, Fig 3). The averted fraction was 31.4% (range by year 7.2–36.7%). Delivery of influenza vaccine through annual vaccination campaigns in March-April (May-June in 2010) with 34% coverage might have averted 35,650 illnesses, 4635 medical visits, 206 influenza-associated hospitalizations and 30 deaths in pregnant women over the 5-year study period. The averted fraction was 12.8% (range by year: 6.1–22.2%). Delivery of influenza vaccine through semiannual vaccination campaigns in March-April (May-June in 2010) and Sept-Oct achieving overall coverage of 69% might have averted 82,719 illnesses, 10,753 medical visits, 478 influenza-associated hospitalizations and 69 deaths in pregnant women over the 5-year study period (Fig 3). The averted fraction was 29.6% (range by year 9.0–38.1%).

#### Infant outcomes averted

Delivery of influenza vaccine through routine antenatal care with 69% coverage might have averted 34,692 illnesses, 14,397 medical visits, 927 influenza-associated hospitalizations, and 123 deaths in infants aged 0–5 months over the 5-year study period (Table 3, Fig 3). The averted fraction in infants aged 0–5 months was 22.1% (range by year 4.1–37.8%). Delivery of influenza vaccine through annual vaccination campaigns in March-April (May-June in 2010) with 34% coverage might have averted 18,257 illnesses, 7576 medical visits, 488 influenza-associated hospitalizations and 69 deaths in infants aged 0–5 months over the 5-year study period. The averted fraction was 11.6% (range by year 3.9–19.9%). Delivery of influenza vaccine through semiannual vaccination campaigns in March-April (May-June in 2010) and Sept-Oct achieving overall coverage of 69% might have averted 34,629 illnesses, 14,371 medical visits, 925 influenza-associated hospitalizations and 126 deaths in infants aged 0–5 months over the 5-year study period. The averted fraction was 22.0% (range by year 5.3–37.7%).

#### Number needed to vaccinate

The number needed to vaccinate to prevent one maternal or infant hospitalization was similar for all three strategies (ANC– 3933, annual campaign– 4004 and semiannual campaigns– 4020). Likewise, the number needed to vaccinate to prevent one maternal or infant death was similar for all three strategies (ANC– 28,776, annual campaigns– 28,072 and semiannual campaigns– 28,923).

### Sensitivity analysis assuming higher risk of influenza-associated hospitalization in pregnant women

The scenarios described above estimate influenza-associated hospitalization rates from women aged 15–49 years and assume no increased risk of influenza-associated hospitalization in pregnant women. Combining data from 3 surveillance systems in Kenya, Malawi and South Africa, and weighting according to the number of influenza-positive pregnant women enrolled, we found that pregnant women in sub-Saharan Africa have increased risk of influenza-associated hospitalization, odds ratio (OR) = 2.9 (95% CI 1.0–12.3) [30]. Because our model uses hospitalizations as the base for determining rates of other outcomes, using a higher rate of influenza-associated hospitalization in pregnant women in our model caused proportionate increases in all illness types that we felt were not valid. Using a higher rate of hospitalization in pregnant women did not alter the annual averted fraction of illness for both pregnant women and infants; however, it did decrease the overall averted fraction of illness because of the increased proportion of illness during the 2010 season when vaccine was not available until May. This did not change the findings of the comparisons between different vaccine distribution strategies.

## Discussion

Over a five year period, we estimated that influenza caused thousands of respiratory hospitalizations among pregnant women and their young infants. Young infants had particularly high rates of influenza-associated hospitalization. Regardless of the immunization strategy, if 55% of pregnant women received influenza vaccination during pregnancy approximately 20% of influenza-associated illness in pregnant women and 17% in young infants could be averted. In all scenarios, the averted fraction of influenza-associated illness was lower in young infants than for pregnant women; however, due to the high rates of severe disease in young infants more hospitalizations and deaths are averted in infants than pregnant women through maternal influenza vaccination.

In this model, there were no significant differences in the proportion of maternal or infant illnesses averted through different influenza vaccine delivery mechanisms when vaccine coverage was held constant (55%). With different assumptions about vaccine coverage, a higher proportion of maternal and infant influenza-associated illness may have been prevented through delivery of influenza vaccine in antenatal care or semiannual campaigns than in annual campaigns, although confidence limits overlap. The only study to examine the feasibility of vaccinating year-round in antenatal care found that the vaccine prevented influenza-like illness in pregnant women and laboratory-confirmed influenza-associated illness in infants aged 0–5 months [33].

Policy makers in resource limited countries may find annual campaigns the most effective means of protecting pregnant women and young infants if there are insufficient doses of influenza vaccine to achieve >30% coverage. A single annual campaign will likewise reduce the administrative burden of distributing vaccine throughout the year via ANC or distribution twice annually for semiannual campaigns. Because of the relatively short shelf-life of influenza vaccines and antigenic drift in circulating influenza viruses, it is essential to use the most up-to-date vaccine available to confer protection to mother and infant. Offering influenza vaccine throughout the year in ANC or via semiannual campaigns would require coordinated distribution of the new season’s vaccine along with timely removal of any remaining doses of the prior season’s vaccine. Policy makers will also need to consider facility-level cold chain capacity and local differences in antenatal care-seeking behaviors to determine the optimal distribution strategy in their country. With limited cold chain capacity it might be necessary to offer influenza vaccination over a longer period of time than a single 2 month campaign. Likewise if women receive few antenatal care visits and/or present late for antenatal care it may be useful to offer influenza vaccination over a longer period of time than a single 2 month campaign in order to improve vaccine coverage. In some cultures, influenza vaccination may also serve as an inducement to seek antenatal care if offered as part of a focused antenatal care package.

There were several limitations to this static model for assessing the impact of different modes of influenza vaccine delivery in antenatal care. First, the model did not adjust for vaccine effectiveness or disease severity by circulating strain type, but used the same estimates across all years due to limited available data. The pandemic strain was particularly severe among pregnant women compared to the previous seasonal influenza A(H1N1) virus and years where influenza A(H3N2) is the predominant circulating virus typically result in more influenza-associated hospitalizations and deaths. Likewise, vaccine effectiveness against influenza A(H3N2) may not be as high as effectiveness against influenza A(H1N1) or influenza B viruses. Virus-specific vaccine effectiveness may alter the overall impact of influenza vaccine on health outcomes for pregnant women and their infants; however, it would not impact the relative outcomes by scenario. Second, we assumed that vaccine effectiveness against laboratory-confirmed influenza hospitalization and death were the same as vaccine effectiveness against laboratory-confirmed influenza illness. It is possible that vaccine effectiveness is higher or lower against severe disease and death than against mild disease; however, studies of vaccine effectiveness are generally not powered to measure effectiveness against severe disease. Third, we assumed protection conferred through immunization or natural infection would be complete and last for the first 6 months of an infant’s life. More recent data suggest that this protection may be limited to the first 4 months of life. Fourth, our surveillance only assessed acute respiratory illness in infants. Young infants with influenza infection may be less likely to present with respiratory symptoms than older children so this may have led to an underestimation of the burden of influenza-associated illness in infants aged 0–5 months. A study in Finland found that nearly half of all influenza-positive infants aged 0–5 months were admitted with a non-respiratory diagnosis [34]. Furthermore, we did not adjust our hospitalization rates to account for the increased risk of influenza-associated hospitalization in pregnant women compared to non-pregnant women which may have underestimated the potential impact of maternal influenza vaccination on maternal hospitalizations and deaths.

This approach only estimates the direct effects of vaccination to vaccinated women and their live-born infants, and does not reflect any potential indirect impact of vaccination (the additional outcomes that may have been averted by decreasing the overall disease infectivity through vaccination) or the potential impact of vaccination on disease severity, birth weight, preterm birth, or fetal loss. Another limitation is that many of the assumptions in this model are generated from surveillance of relatively small populations. Assumptions from these small populations about the proportion of outpatient vs. inpatient illnesses, timing of outpatient vs. inpatient illnesses, healthcare seeking for respiratory illness, and the proportion of deaths occurring in the community may not be representative of respiratory illnesses and deaths throughout Kenya. And finally, we assumed that semiannual campaigns delivered over 4 months annually could achieve the same vaccine coverage as year-round antenatal care distribution. It is likely that distribution throughout the year might achieve higher coverage than intermittent campaigns but without recent data on maternal vaccination campaigns in Kenya it is difficult to predict the anticipated coverage from these different strategies.

## Conclusions

Over the 5-year period of study, depending on the mode of distribution and the vaccine coverage achieved, 12.8–31.4% of influenza-associated illnesses, medical visits, hospitalizations and deaths among pregnant women and 11.6–22.1% of influenza-associated illnesses, medical visits, hospitalizations and deaths among infants aged 0–5 months might have been prevented through maternal influenza immunization. Each of the proposed strategies for delivery of influenza vaccine to pregnant women has advantages and disadvantages in terms of infrastructure requirements (cold chain, delivery systems, personnel, etc.) and associated costs. Governments considering introduction of influenza vaccination for pregnant women will have to assess existing infrastructure for vaccine delivery and determine the best vaccine distribution method for their country. Additional analysis of the cost and cost-effectiveness of different methods of vaccine delivery to pregnant women will be important to inform policy decisions in Kenya and other tropical countries where influenza circulation is not confined to a single seasonal peak.
